# Compositional and Metabolomic Characterization of Major On-Farm Side Streams and Derived Products of Industrial Fine-Flavor Cocoa Processing

**DOI:** 10.1021/acsomega.6c06084

**Published:** 2026-09-30

**Authors:** Daniela Burgos-Toro, Ana M. León-Inga, María J. Chica, José M. Guillamón, Mónica P. Cala, Miguel Fernández-Niño

**Affiliations:** † Grupo de Sistemas de Producción Sostenibles/Sustentables (SISPROS), Facultad de Ciencias Agropecuarias, 268973 Universidad de Cundinamarca, Fusagasugá 252212, Colombia; ‡ MetCore - Metabolomics Core Facility, Vice-Presidency for Research, Universidad de Los Andes, Bogotá 111711, Colombia; § CasaLuker S.A., Manizales 170004, Colombia; ∥ Department of Biotechnology, Institute of Agrochemistry and Food Technology (IATA-CSIC), Paterna 46980, Spain

## Abstract

The fine-flavor cocoa agro-industrial chain generates on-farm side streams with distinct origins and processing histories, yet their compositional diversity remains insufficiently characterized under industrial conditions. Here, we performed an integrated compositional and metabolomic characterization of cocoa pod husk, cocoa pulp juice, and cocoa syrup generated within a large-scale monoclonal fermentation system (Luker 40; ∼500 kg per fermentation box) operating within an industrial fine-flavor cocoa production system exceeding 73,000 tons annually. Metabolomic characterization was performed using three independent industrial production batches as biological replicates (n = 3), whereas proximate and mineral analyses were performed on composite samples prepared from the three batches. Mineral composition showed the largest relative differences among matrices, with cocoa syrup reaching an ash content approximately 12 times that of cocoa pulp juice. Complementary LC-MS and GC-MS analyses enabled the annotation of 82 metabolites (46 by LC-MS and 36 by GC-MS), revealing distinct matrix-resolved profiles, including caffeic acid, epicatechin, and procyanidin B1 in cocoa pod husk; glutamine, glutathione, and indole carboxaldehyde in cocoa pulp juice; and N-(deoxyfructosyl) amino acid derivatives in cocoa syrup. To our knowledge, this is the first joint compositional and metabolomic description of a thermally concentrated, fermentation-derived cocoa syrup together with prefermentation cocoa pulp juice recovered from the same production batches. These results provide a compositional baseline for subsequent targeted quantification, extraction/recovery, and functional validation, supporting the rational prioritization of cocoa side streams and derived products for future valorization studies.

## Graphical abstract

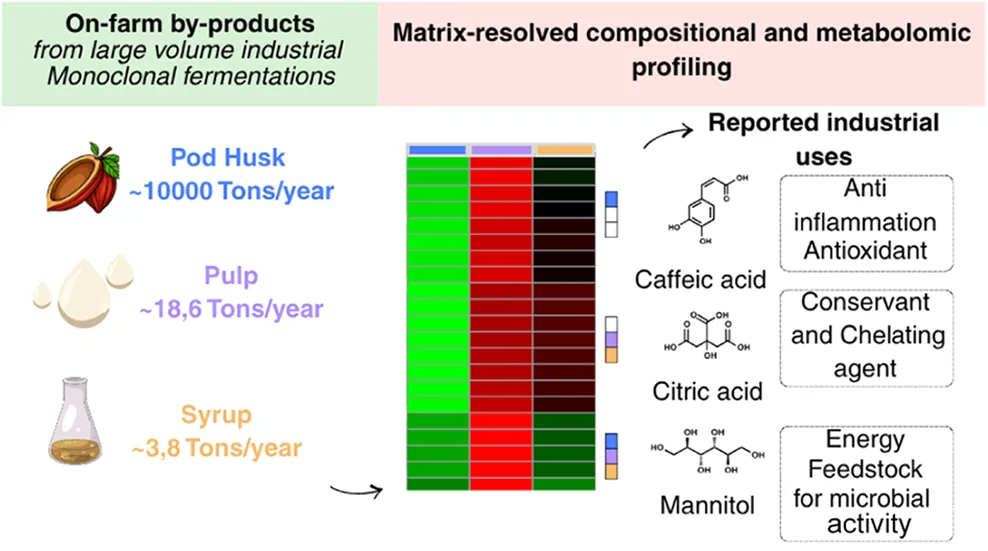

## Introduction

The cultivation of cacao (*Theobroma cacao L*.) has significant economic, social, and cultural relevance at the global level, driven both by the sustained growth of chocolate consumption and by the consolidation of specialized value chains. In 2023, global production reached nearly 5 million tons, with Africa as the main producing region (73.4%), followed by Latin America and the Caribbean (20%).^1^ In the Latin American context, cacao has also played a strategic socio-cultural role in rural development dynamics and territorial strengthening; in countries such as Colombia, it has been promoted as a productive alternative in processes of substitution of illicit economies and reconstruction of the agricultural fabric in postconflict scenarios.^2,3^ In parallel, several countries in the region have consolidated the production of fine-flavor cocoa because of domestication and selection processes that have made it possible to obtain differentiated and competitive sensory profiles in specialized market niches.^4,5^ Despite this relevance, only a small fraction of the cocoa fruit is ultimately used for chocolate production, while most of the biomass remains underutilized, representing a largely unexplored resource for the development of new value-added ingredients and sustainable product streams.^6^

During cocoa processing, different on-farm side streams and derived products are generated at clearly differentiated stages of postharvest handling. During fruit opening, the beans are separated, and the cocoa pod husk is generated, constituting the main unavoidable solid residue of this initial stage. Subsequently, before the beginning of fermentation, part of the pulp (mucilage) surrounding the beans may be removed as a liquid fraction, hereafter referred to as cocoa pulp juice, as a practice commonly implemented in industrial systems to control the fermentation process.^7^ Finally, during fermentation, the exuded liquids known as sweatings are generated, resulting from the drainage of the mucilage, which may be recovered and subjected to thermal treatment to control additional microbial activity, giving rise to products such as cocoa syrup in a process currently employed by the company CasaLuker S.A.S. This differentiation is relevant since these materials do not belong to a single category of waste nor are they generated under equivalent conditions. The term byproduct is used throughout, in this broad sense, encompassing both unavoidable residues and materials that have already undergone a first processing step, rather than as a synonym for waste. Instead, they represent distinct process-derived streams within on-farm industrial postharvest systems, each with specific origins, degrees of intentionality, and technological handling.

While the cocoa pod husk is generated as a structural residue associated with the opening of the fruit, the juice from the cocoa pulp represents a liquid fraction deliberately recovered before fermentation, and cocoa syrup is the product of an initial valorization stage that has already been applied to the fermentation exudates. Thus, the three materials occupy different positions along the same processing chain, ranging from the unavoidable residue to the deliberately recovered fraction and the stabilized derivative product, and are included here precisely because this gradient has not been previously characterized within the same production system.

In terms of yield, only between 5% and 10% of the fruit corresponds to the beans used for chocolate production, while the remaining 90–95% is distributed among the cocoa pod husk, pulp, and bean shell.^8–11^ The cocoa pod husk represents approximately 70–80% of the total fruit volume, followed by the pulp and bean shell, which account for around 20%.^12–15^ This disproportion highlights the relevance of these byproducts as abundant, yet underexploited, raw materials with potential for valorization within circular and sustainable cocoa production systems. When these materials are not properly managed, their direct disposal may favor the proliferation of pests and pathogens and contribute to greenhouse gas emissions such as methane and carbon dioxide.^16,17^

Studies have shown that these byproducts present complex chemical compositions. The cocoa pod husk and the bean shell contain high levels of fiber, protein, and lignocellulosic components, as well as lipid fractions with saturated, monounsaturated, and polyunsaturated fatty acids,^16,18^ in addition to volatile compounds, polyphenols, and methylxanthines.^11,19^ The pulp contains high concentrations of methylxanthines and phenolic compounds,^16,20^ while sweatings are characterized by high contents of soluble carbohydrates and minerals^13^ and have been explored in food applications.^12,20^ However, although there is a growing number of studies on cocoa-derived materials, many focus on isolated components or on small-scale experimental systems.^22–25^ Comparative studies integrating multiple on-farm byproducts under real industrial-scale postharvest conditions remain limited, particularly in large-scale monoclonal fermentation systems.

Particular terminological ambiguity surrounds the liquid fractions generated during this processing chain. The translucent, sugar-rich liquids recovered before or during cocoa fermentation have been variously described as cocoa honey, cocoa sweat, cocoa exudate, and cocoa pulp juice, with these designations often used interchangeably.^13,22^ This interchangeable terminology obscures process-relevant distinctions among materials recovered at different stages and by different means, including juice mechanically separated from the pulp before fermentation, exudates drained from the fermenting mass as pulp depectinization progresses, and products subsequently obtained from these liquid streams through further processing. Existing characterizations of cocoa honey and cocoa sweat have been predominantly physicochemical, reporting soluble solids, pH, titratable acidity, reducing sugars, pectin, and mineral composition,^22,26,27^ while applied studies have evaluated these fractions as fermentation substrates, beverage bases, sourdough ingredients, and additives during bean fermentation.^21,28,29^ More recently, cocoa pulp juice concentrate has been combined with the pectin-rich pod endocarp to produce a sweetening gel capable of replacing refined sugar in chocolate formulations, further illustrating the technological relevance of these liquid fractions beyond fermentation-related uses.^30^ Thus, these overlapping designations encompass materials that may differ in their stage of recovery, processing history, and status as unprocessed process streams or defined industrial products.

These distinctions are also analytically relevant. Untargeted metabolomic descriptions of cocoa liquid fractions remain scarce, particularly for materials subjected to processing after recovery. To our knowledge, no study has jointly characterized a thermally concentrated product derived from fermentation exudates, such as the syrup examined here, and the prefermentation pulp juice recovered from the same production batches. These two materials represent distinct process-defined streams: the juice is recovered before microbial fermentation, whereas the syrup is derived from exudates generated during fermentation and subsequently subjected to thermal stabilization and concentration. Their compositional equivalence, therefore, cannot be assumed from studies of fresh or pasteurized cocoa honey, or other cocoa-derived liquid fractions, supporting the need for separate characterization.

Taken together, three gaps emerge in the available literature. First, cocoa by-products have rarely been comparatively characterized within the same production system and using a common analytical framework, limiting the extent to which differences reported across independent studies can be attributed to the materials themselves rather than to production or analytical variability. Second, overlapping terminology for cocoa liquid fractions often fails to distinguish materials according to their recovery stages and subsequent processing history. Third, untargeted metabolomic information on these process-defined liquid streams remains limited, with no joint characterization, to our knowledge, of prefermentation pulp juice and a thermally concentrated, fermentation-exudate-derived product recovered within the same industrial production batches.

In this context, it is pertinent to generate integrated information that combines proximate analyses and metabolomic approaches to comparatively describe the on-farm byproducts generated at different stages of industrial fine-flavor cocoa processing. The present study was conducted using the fine-flavor cocoa clone Luker 40, cultivated and processed within a large-scale industrial production system associated with one of the world’s major fine-flavor cocoa networks, encompassing more than 73,000 tons of cocoa produced annually, approximately 18,587 ha under sustainable management, and over 15,000 farming families. This framework provides a highly standardized and traceable model for the study of industrial cocoa byproducts generated under real postharvest conditions. Therefore, the present study aimed to perform an integrated compositional and metabolomic characterization of three materials generated at different stages of industrial fine-flavor cocoa processing: cocoa pod husk, cocoa pulp juice, and a syrup obtained by thermal treatment of fermentation sweatings. By jointly examining an unavoidable solid residue, a deliberately recovered prefermentation liquid fraction, and a processed product derived from fermentation exudates, this study captures compositionally distinct streams occupying different positions along the same industrial postharvest chain. Rather than demonstrating direct functional applications, this work provides a compositional basis that may support future studies oriented toward process optimization, targeted extraction, and possible utilization strategies within circular cocoa production systems.

## Materials and Methods

### Source and Sampling of On-Farm Side Streams and Derived Products

Three cocoa-derived matrices were obtained from industrial fine-flavor cocoa processing carried out by CasaLuker S.A. at the Villa Nueva Farm (Casanare, Colombia; 4°35′35.4“N, 72°49′34.3”W), under standard production conditions. The processing system is based on large-scale monoclonal fermentations performed in wooden boxes with a capacity of approximately 500 kg per fermentation batch. Fermentation containers, collection vessels, and transfer lines are food-grade materials managed under the certified quality system of the producing company, and no mineral additives are used at any stage of the post-harvest chain. The analyzed by-products were generated at different stages of the on-farm post-harvest processing chain (Figure 1).

**Figure 1:**
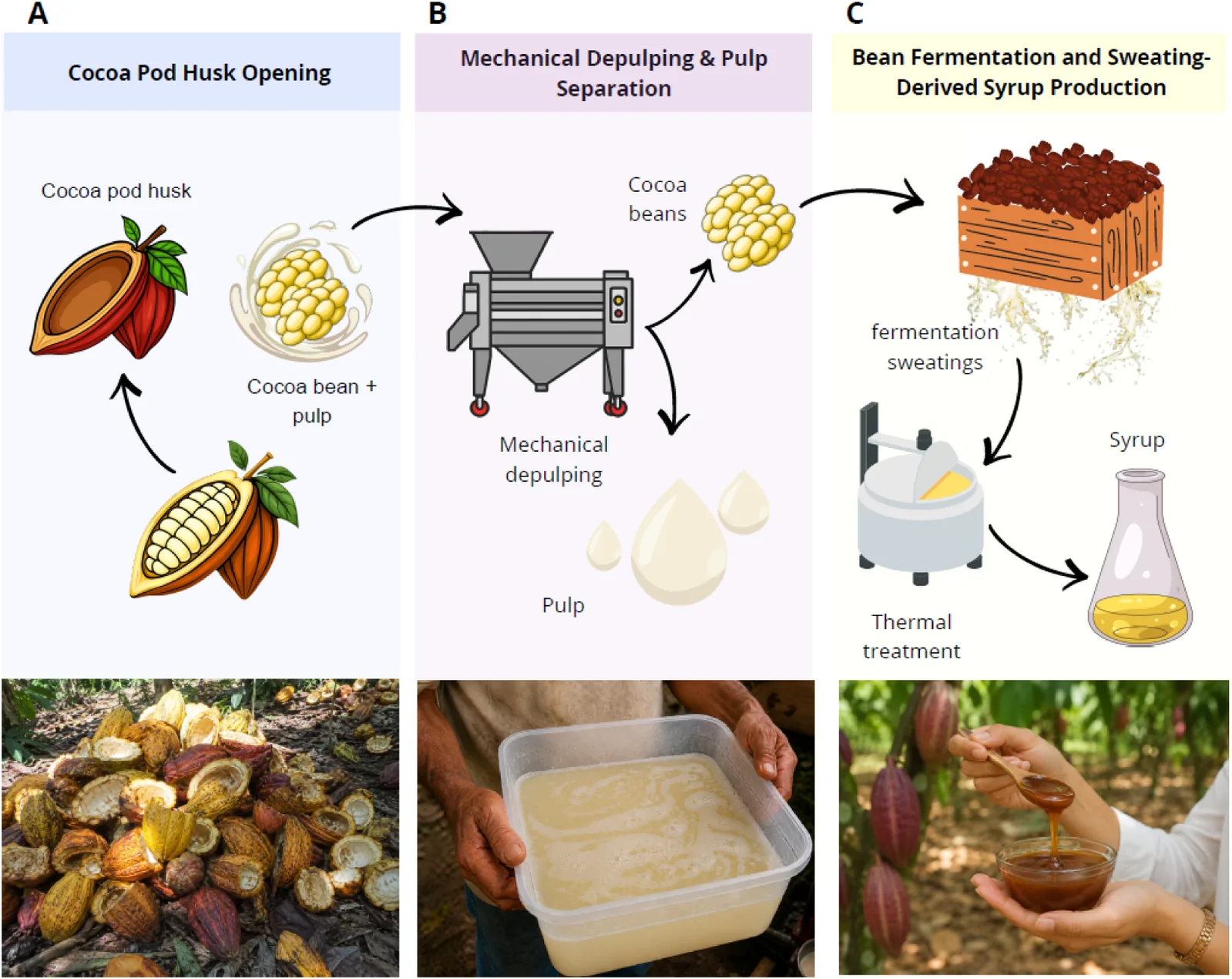
Industrial generation of on-farm cocoa byproducts during fine-flavor cocoa processing. (A) Cocoa pod husk generated during fruit opening, including the intact pod, mechanical opening, and the resulting chipped husk. (B) Cocoa pulp juice deliberately recovered prior to fermentation through mechanical depulping, corresponding to the liquid fraction of the mucilage. (C) Cocoa syrup obtained from fermentation sweatings, including fermentation in wooden boxes, collection of exudates, and subsequent thermal treatment to stabilize the product. The three photographic-style images shown at the bottom of panels A–C were generated using artificial intelligence and are included for illustrative purposes only.

Cocoa pod husk is obtained during fruit opening and represents the main unavoidable solid residue of this stage (∼10,000 tons per year), accounting for approximately 74% of the fruit mass. Cocoa pulp juice corresponds to the liquid fraction of the mucilage intentionally removed prior to fermentation through mechanical depulping as part of routine industrial practices aimed at ensuring homogeneous fermentation, with an approximate annual yield of 16.8 tons. The third matrix, cocoa syrup, is obtained from fermentation exudates, that is, the liquids exuded from the fermenting mass throughout the process. These exudates were collected and subjected to a controlled heat treatment applied by CasaLuker S.A. to halt microbial activity and stabilize the material, resulting in a shelf-stable viscous syrup produced at an approximate rate of 3.8 tons per year. The specific parameters of this treatment constitute confidential industrial technical information; therefore, the material was characterized as delivered by the production system, in the form in which it is generated at an industrial scale. Its viscous consistency necessitated gravimetric sampling rather than volumetric sampling in all analyses.

Samples were collected from three independent routine industrial production batches of the same clone (Luker 40), processed in different months, thereby capturing temporal variability in industrial production while maintaining a constant genetic background. Each production batch constituted one independent biological replicate for each matrix. For each batch, cocoa pod husk material from three different pods was pooled and homogenized under liquid nitrogen to obtain a single representative sample, while cocoa pulp juice and cocoa syrup were collected as homogeneous samples at their corresponding stages of the processing chain. This resulted in three independent biological replicates per matrix (n = 3) for metabolomic characterization. All samples were transported to the laboratory on dry ice and stored at −80 °C until analysis. For proximate and mineral characterization, equal amounts of material from the three production batches were pooled into a single composite sample for each byproduct, providing an integrated compositional reference for each matrix.

### Compositional Analysis

Proximate and mineral analyses were performed by LASEREX (Laboratorio de Servicios de Extensión en Análisis Químicos, Universidad del Tolima, Colombia) in accordance with standard AOAC procedures. Crude protein content was determined using the Kjeldahl method, while ash and ether extract were quantified using gravimetric methods. Mineral elements (calcium, magnesium, sodium, potassium, iron, copper, manganese, zinc, boron, phosphorus, and sulfur) were determined by spectrophotometric techniques, including atomic absorption and UV–vis, depending on the element analyzed. For each byproduct, a composite sample prepared by pooling equal amounts of material from the three independent production batches was analyzed in triplicate (n = 3). Accordingly, the reported dispersion represents analytical repeatability rather than biological variability. All compositional values are reported on a fresh-weight basis. Moisture and dry matter were not determined; consequently, values cannot be recalculated on a dry-weight basis. Analyses were performed by an external service laboratory operating under an ISO 9001:2015-certified quality management system. Method-specific limits of detection, limits of quantification, and recovery data were not provided in the analytical reports available for this study. All analytes yielded values within the laboratory’s reportable range.

Fiber was outside the analytical scope adopted here, which focused primarily on the metabolomic characterization of soluble and extractable compounds. Potentially toxic elements, including cadmium, lead, arsenic, and mercury, were likewise not determined, and, therefore, this study does not constitute a safety assessment of the analyzed byproducts.

### Metabolite Characterization of On-Farm Cocoa Side Streams and Derived Products through LC-MS Analysis

#### Sample Preparation and LC-MS Analysis

For LC-MS analysis, 10 μL of cocoa pulp juice and 10 mg each of cocoa pod husk and cocoa syrup were used; the latter was weighed due to its viscous nature. Each sample was extracted with 400 μL of H_2_O:MeOH (75:25, v/v), followed by vortex mixing at 3200 rpm for 15 min and sonication for 10 min. Samples were then centrifuged at 14,000 rpm and 4 °C for 10 min. The supernatants were filtered through 0.22 μm PTFE filters prior to analysis.

Chromatographic separation was performed using an Agilent 1260 liquid chromatography system coupled to a 6545 Quadrupole Time-of-Flight (Q-TOF) mass analyzer with an electrospray ionization (ESI) source. Source parameters were set as follows: capillary voltage (3000 V), drying gas temperature (325 °C), drying gas flow (8 L/min), nebulizer pressure (50 psi), sheath gas temperature (350 °C), sheath gas flow (11 L/min), fragmentor voltage (175 V), skimmer voltage (65 V), and octopole RF (750 V). A 10 μL aliquot of each sample was injected into an InfinityLab Poroshell 120 EC-C18 column (3.0 × 100 mm, 2.7 μm) at 30 °C, operated at a flow rate of 0.4 mL/min. The mobile phase consisted of (A) water with 0.1% (v/v) formic acid and (B) acetonitrile with 0.1% (v/v) formic acid. The gradient started at 2% B, increased to 30% over 15 min, then to 98% at 17 min, was held for 3 min, and finally returned to the initial conditions for re-equilibration. Mass detection was performed in both positive and negative ionization modes under full scan and Auto MS/MS acquisition over a mass range of *m*/*z* 100–1700 at a rate of 3 spectra/s. MS/MS spectra were acquired using collision energies ranging from 20 to 40 eV. Data acquisition was performed using Agilent MassHunter Data Acquisition software (version 10.0). Continuous mass correction was applied using reference ions at *m*/*z* 121.0509 [C_5_H_4_N_4_]^+^ and *m*/*z* 922.0098 [C_18_H_18_O_6_N_3_P_3_F_24_]^+^ in positive mode, and *m*/*z* 112.9856 [C_2_H_4_O_2_NF_3_-(NH_4_)]^−^ and *m*/*z* 1033.9881 [(C_18_H_18_O_6_N_3_P_3_F_24_ + trifluoroacetic acid)-H]^−^ in negative mode.

### Data Processing and Compound Annotation

Data were processed using Agilent MassHunter Profinder 10.0 software with the Recursive Molecular Feature Extraction algorithm. Features were filtered using solvent blanks, and relative abundance was calculated for each biological replicate by dividing the peak area of each feature by the total peak area of the corresponding sample. For the LC-MS data set, to ensure high MS/MS spectral quality and minimize false-positive annotations, only features with relative abundance above 1.0% were selected for compound identification. The identification process was carried out considering monoisotopic patterns, adduct formation, molecular formulas, and MS/MS fragmentation. Databases including METLIN, KEGG, LIPID MAPS, and HMDB were used for compound assignment. Identification levels were assigned according to Blaženović and collaborators,^31^ who established increasing levels of confidence, including level 4 (exact-mass match/unknown feature), level 3 (possible structure, confirmation with molecular formula), level 2 (likely structure or consistency with literature spectra), level 1 (a confident 2D structure or confirmation with in-house standards), and level 0 (an unambiguous 3D structure). Caffeic acid, the only metabolite assigned to identification level 1, was confirmed by comparison with an authentic standard analyzed under the same experimental conditions.

### Metabolite Characterization of On-Farm Cocoa Side Streams and Derived Products through GC-MS Analysis

#### Sample Preparation and GC-MS Analysis

For GC-MS analysis, 100 μL of cocoa pulp juice and 5 mg each of cocoa pod husk and cocoa syrup were used, with the syrup weighed due to its viscous nature. Samples were extracted with 200 μL of methanol, followed by vortex-mixing for 5 min and sonication for 10 min. The extracts were then centrifuged at 15,000 rpm for 10 min at room temperature. A 50 μL aliquot of the supernatant was subjected to a two-step derivatization procedure. First, methoximation was performed by adding 20 μL of O-methoxamine hydrochloride in pyridine (15 mg/mL) and incubating under appropriate conditions. Subsequently, silylation was carried out using 20 μL of N,O-bis­(trimethylsilyl)­fluoroacetamide (BSTFA) with 1% trimethylchlorosilane (TMCS). Prior to GC-MS analysis, 80 μL of an internal standard solution (C18) was added to each sample. Chromatographic analysis was performed using an Agilent 7890B gas chromatograph coupled to an Agilent quadrupole time-of-flight (Q-TOF-7250) mass detector. Samples were introduced through a split/splitless injector operated in split mode (250 °C, split ratio 1:30) with an automatic injector (Agilent Technologies 7693A). Separation was achieved using an Agilent J&W HP-5MS column (30 m × 0.25 mm × 0.25 μm), with helium as the carrier gas at a constant flow rate of 0.7 mL/min. The oven temperature program started at 60 °C (held for 1 min), increased at 10 °C/min to 325 °C, and was held for 10 min. The temperatures of the transfer line to the detector, the ion source filament, and the quadrupole were maintained at 280 °C, 230 °C, and 150 °C, respectively. Electron ionization (EI) was performed at 70 eV with a filament emission current of 5 μA. Automated mass calibration and tuning (Autotune) were conducted prior to the analytical sequence. Mass spectra were acquired at a rate of 5 scans per second over a mass range of *m*/*z* 50–600. Data acquisition was performed using Agilent MassHunter Data Acquisition software (version 10.0).

#### Data Processing and Compound Identification

GC-MS data were processed with Agilent MassHunter Unknowns Analysis B.10.00 software, employing the SureMass peak-detection strategy. Solvent blanks were included in the analytical sequence and used to exclude background signals. Metabolite identification was performed by comparison against the Fiehn GC-MS Metabolomics RTL Library (version 2011) and the NIST library (version 2017), considering mass spectral matching (match factor >90) and retention behavior. No relative abundance threshold was applied to the GC-MS data set; annotation was governed by the spectral match factor and retention index criteria described above, and features without a library match were not annotated or reported. Retention indices (RI) were calculated using external standards, including fatty acid methyl esters (FAMEs) and *n*-alkanes (C7–C20), to support compound annotation. Metabolite identification was based on spectral similarity, retention time alignment, and RI matching. Relative abundances were obtained using total peak area normalization, where each peak area was divided by the total peak area of the corresponding sample.

### Statistical Analysis and Data Visualization

For compositional characterization, data are reported as mean ± standard deviation of three analytical determinations performed on one composite sample per byproduct. Because these determinations represent analytical replicates rather than independent biological units, no inferential statistical comparisons among matrices were performed. Analytical repeatability was evaluated for each parameter and matrix using the coefficient of variation (CV), calculated as (SD/mean) × 100. Complete analytical repeatability data are provided in Supplementary Table S1.

For metabolite characterization, the three independent production batches constituted the biological replicates (n = 3 per matrix). Relative metabolite abundances were calculated for each biological replicate by normalizing the individual peak areas to the total peak area of the corresponding sample. The nine normalized profiles (three biological replicates per matrix) were uploaded to MetaboAnalyst 6.0 (https://www.metaboanalyst.ca/MetaboAnalyst/) for heat map generation, with the three biological replicates averaged for visualization at the matrix level. The reported relative abundances likewise correspond to the average of the three biological replicates. No additional analytical replicates were performed for the metabolomic characterization. Pooled QC samples were not included in the analytical sequences; accordingly, a QC-based signal-drift correction was not applied.

## Results and Discussion

### Cocoa By-Products Show Similar Proximate Composition but Differ in Mineral Content

Three cocoa byproducts generated at successive stages of on-farm fine-flavor cocoa processing were characterized under industrial conditions (Figure 1): cocoa pod husk, cocoa pulp juice, and cocoa syrup. Proximate composition was comparable across the three matrices, whereas mineral content differed by up to 2 orders of magnitude (Table 1).

**Table 1:** Proximate Composition and Mineral Content of Cocoa By-Products Generated during On-Farm Fine-Flavor Cocoa Processing^a^

Parameter	Unit	Cocoa Pod Husk	Cocoa Pulp Juice	Cocoa Syrup
*Proximate composition*				
Crude protein	%	1.223 ± 0.023	0.860 ± 0.010	1.257 ± 0.031
Ether extract	%	0.307 ± 0.006	0.024 ± 0.001	0.110 ± 0.010
Ash	%	2.010 ± 0.026	0.433 ± 0.025	5.383 ± 0.012
*Macrominerals*				
Calcium (Ca)	%	1.020 ± 0.010	0.058 ± 0.002	0.253 ± 0.006
Potassium (K)	%	0.450 ± 0.010	0.092 ± 0.003	1.257 ± 0.032
Magnesium (Mg)	%	0.049 ± 0.001	0.015 ± 0.001	0.083 ± 0.002
Phosphorus (P)	%	0.044 ± 0.001	0.010 ± 0.000	0.035 ± 0.001
Sulfur (S)	%	0.023 ± 0.001	0.010 ± 0.000	0.026 ± 0.001
Sodium (Na)	mg/kg	107.83 ± 1.02	38.30 ± 0.10	364.76 ± 0.90
*Microminera/ls*				
Boron (B)	mg/kg	79.03 ± 0.96	6.64 ± 0.24	44.30 ± 0.36
Iron (Fe)	mg/kg	58.40 ± 0.20	3.11 ± 0.08	29.80 ± 1.56
Manganese (Mn)	mg/kg	38.90 ± 0.10	3.67 ± 0.01	15.54 ± 0.46
Zinc (Zn)	mg/kg	27.03 ± 0.21	4.52 ± 0.01	17.59 ± 0.04
Copper (Cu)	mg/kg	13.13 ± 0.06	1.73 ± 0.04	5.66 ± 0.01

a Values are expressed on a fresh weight basis as mean ± standard deviation of three analytical replicates (N = 3) of one composite sample per by product prepared from three independent industrial production batches, and the reported dispersion therefore represents analytical repeatability . Crude protein was determined by the Kjeldahl method; ash and ether extract by gravimetric methods ; Ca, Mg, Na, K, Fe, Cu, Mn, and Zn by atomic absorption spectrophotometry ; and B, P, and S by UV-Vis spectrophotometry. Analyses were performed by LASEREX (Universidad del Tolima, Colombia) following AOAC procedures.

Because cocoa pulp juice and cocoa syrup originate from the same production batches but are recovered at different stages of the process, their paired characterization reduces raw-material variability and enables direct assessment of their compositional nonequivalence. Crude protein content was low across all matrices, ranging from 0.860% (cocoa pulp juice) to 1.257% (cocoa syrup), with cocoa pod husk showing an intermediate value of 1.223%. These values are below those previously reported for cocoa pod husk (approximately 2.4–4.3%),^11,32^ while remaining consistent with reported protein content in cocoa pulp (∼0.7%).^29^ Similarly, ethereal extract content was uniformly low across all samples (pod husk: 0.307 ± 0.006%; pulp juice: 0.024 ± 0.001%; syrup: 0.110 ± 0.010%). These values are slightly below those reported for cocoa pod husk (>0.5%)^11,32^ and align with previous observations for cocoa pulp,^29^ confirming that these byproducts are characterized by a limited lipid fraction. These values are expressed on a fresh-weight basis, whereas published compositions for cocoa pod husk are generally reported on a dry-weight basis; therefore, direct numerical comparisons should be interpreted with caution. The monoclonal industrial system analyzed here provides a consistent and traceable definition of each matrix, which is what allows the three byproducts to be compared directly with one another under a single analytical protocol.

When compared with protein and lipid fractions, ash content showed pronounced differences among matrices and represented the most evident compositional variation. Cocoa syrup exhibited the highest ash content (5.383 ± 0.012%), followed by cocoa pod husk (2.010 ± 0.026%) and cocoa pulp juice (0.433 ± 0.025%). The ash content observed for cocoa pod husk is lower than previously reported values (8.8–11.4%),^11,32^ while the values for cocoa pulp juice are consistent with the literature.^29^ The elevated ash content in cocoa syrup likely reflects the release (leaching) of soluble minerals from cocoa tissues during fermentation, followed by their concentration during the collection and thermal stabilization of fermentation-derived liquors. This interpretation is supported by a direct comparison within the current data set: cocoa syrup has an ash content approximately 12 times higher than that of the pulp juice recovered from the same production batches (5.383 ± 0.012% vs 0.433 ± 0.025%). Because both fractions originate from the same production batches, differences associated with genetic background and raw material source are minimized. However, the two matrices differ in their stage of recovery and processing history: cocoa pulp juice is mechanically recovered before fermentation, whereas cocoa syrup derives from exudates collected during fermentation and subsequently subjected to thermal stabilization and concentration. The observed difference is therefore consistent with the combined influence of fermentation-associated mineral mobilization and subsequent processing, supporting the compositional nonequivalence of the two liquid fractions.

This concentration effect also has implications beyond those associated with nutritional minerals. If soluble elements are enriched during the harvesting and thermal stabilization of fermentation liquors, then potentially toxic elements present in the raw material could follow the same pattern. Cadmium is particularly relevant in this context, given its documented accumulation in cocoa grown in Latin American soils and the maximum levels established for cocoa products under Commission Regulation (EU) 2023/915. Therefore, the determination of potentially toxic elements would be a prerequisite for assessing the suitability of these fractions for food use and is particularly relevant for concentrated products, such as the syrup described here.

The mineral profile further reinforced these differences. Cocoa pod husk showed appreciable micromineral concentrations, including iron (58.40 ± 0.20 mg/kg), manganese (38.90 ± 0.10 mg/kg), boron (79.03 ± 0.96 mg/kg), zinc (27.03 ± 0.21 mg/kg), and copper (13.13 ± 0.06 mg/kg). These values are generally consistent with previously reported ranges for this residue, although zinc was notably lower than the 40–54 mg/kg previously described by refs. 11,33 , a difference that may again reflect the nonequivalent basis of comparison, as well as differences in cocoa source, soil mineral availability, and fruit maturity at harvest. Reflecting its liquid and fermentation-derived origin, cocoa syrup showed the highest concentrations of soluble minerals, particularly sodium (364.76 ± 0.90 mg/kg), potassium (1.257 ± 0.032%), and magnesium (0.083 ± 0.002%), consistent with its origin as a concentrated fraction of fermentation-derived liquors. Cocoa pulp juice displayed the lowest overall mineral concentrations, with calcium (0.058 ± 0.002%), magnesium (0.015 ± 0.001%), zinc (4.52 ± 0.01 mg/kg), and copper (1.73 ± 0.04 mg/kg) all markedly below the other matrices, in agreement with previous reports for cocoa pulp.^29^

### Mineral Enrichment in Cocoa Syrup Is Selective and Tracks Ion Mobility in Plant Tissue

This enrichment is not simply the result of concentration. When each element is compared with the overall 12.4-fold increase in ash between the two liquid fractions, only potassium exceeds it (13.7-fold). Iron and sodium follow at 9.6- and 9.5-fold, while the remaining divalent cations fall well below the ash factor (magnesium 5.5, calcium 4.4, manganese 4.2, zinc 3.9, and copper 3.3-fold). Therefore, mineral mobilization during fermentation appears to be selective and broadly consistent with the mobility of each ion in the plant tissue. Potassium is the main free cation in the vacuolar sap and is not structurally bound,^34–36^ so it passes into the exudate as membrane integrity is lost and mucilage degrades.^34,35^ Calcium, magnesium, manganese, zinc, and copper remain partially bound to the pectin network and to the carboxyl groups of phytate and proteins^37^ and are only partially mobilized.^33,36^ Thermal stabilization can further widen this gap as water removal promotes the precipitation of poorly soluble divalent salts. Sodium and iron occupy an intermediate position in this gradient and require more cautious interpretation: sodium is not a significant vacuolar cation in cocoa mucilage, so a contribution from processing water or equipment cannot be ruled out, while the behavior of iron is not fully accounted for by the binding gradient described above. These points remain hypothetical and require experimental verification.

The possibility that the metallic elements originate from the processing system rather than from the plant material can be assessed within the present data set. Cocoa pod husk, which is separated at fruit opening and undergoes essentially no industrial handling, shows the highest concentrations of iron (58.4 mg/kg), manganese (38.9 mg/kg), zinc (27.0 mg/kg), and copper (13.1 mg/kg) of the three matrices, in every case above those of the extensively processed syrup. A contribution from equipment wear would produce the opposite ordering and would not be expected to reproduce a gradient that tracks the binding chemistry of each ion. Moreover, fermentation is carried out in wooden boxes, so the dominant contact material during the stage in which the sweatings are generated is not metallic. Sodium is the single element that departs from this pattern, being higher in the syrup than in the unprocessed husk, which is consistent with the cautionary note above and identifies sodium as the element for which a processing contribution is most plausible.

Overall, proximate composition showed relatively limited variation across cocoa byproducts, whereas mineral profiles exhibited larger relative differences among matrices, with some elements differing by approximately 1 order of magnitude. Within this framework, cocoa syrup could be understood as a mineral-enriched fraction derived from fermentation processes, whereas cocoa pod husk reflects a structurally associated composition, and cocoa pulp juice reflects a more diluted soluble fraction. Importantly, the controlled monoclonal nature of the system analyzed here provides a more defined compositional baseline for these materials, contributing to improved comparability and traceability relative to previous studies. While proximate and mineral analyses capture these broad compositional patterns, subsequent GC-MS and LC-MS analyses provide deeper insight into the metabolic diversity of these byproducts, enabling the identification of volatile and nonvolatile compounds not resolved by conventional approaches.

### Cocoa Side Streams and Derived Products Exhibit Distinct Metabolic Signatures

Heatmaps of the metabolites annotated by LC-MS and GC-MS showed distinct descriptive relative-abundance patterns across the three cocoa matrices (Figure 2A,B).

**Figure 2:**
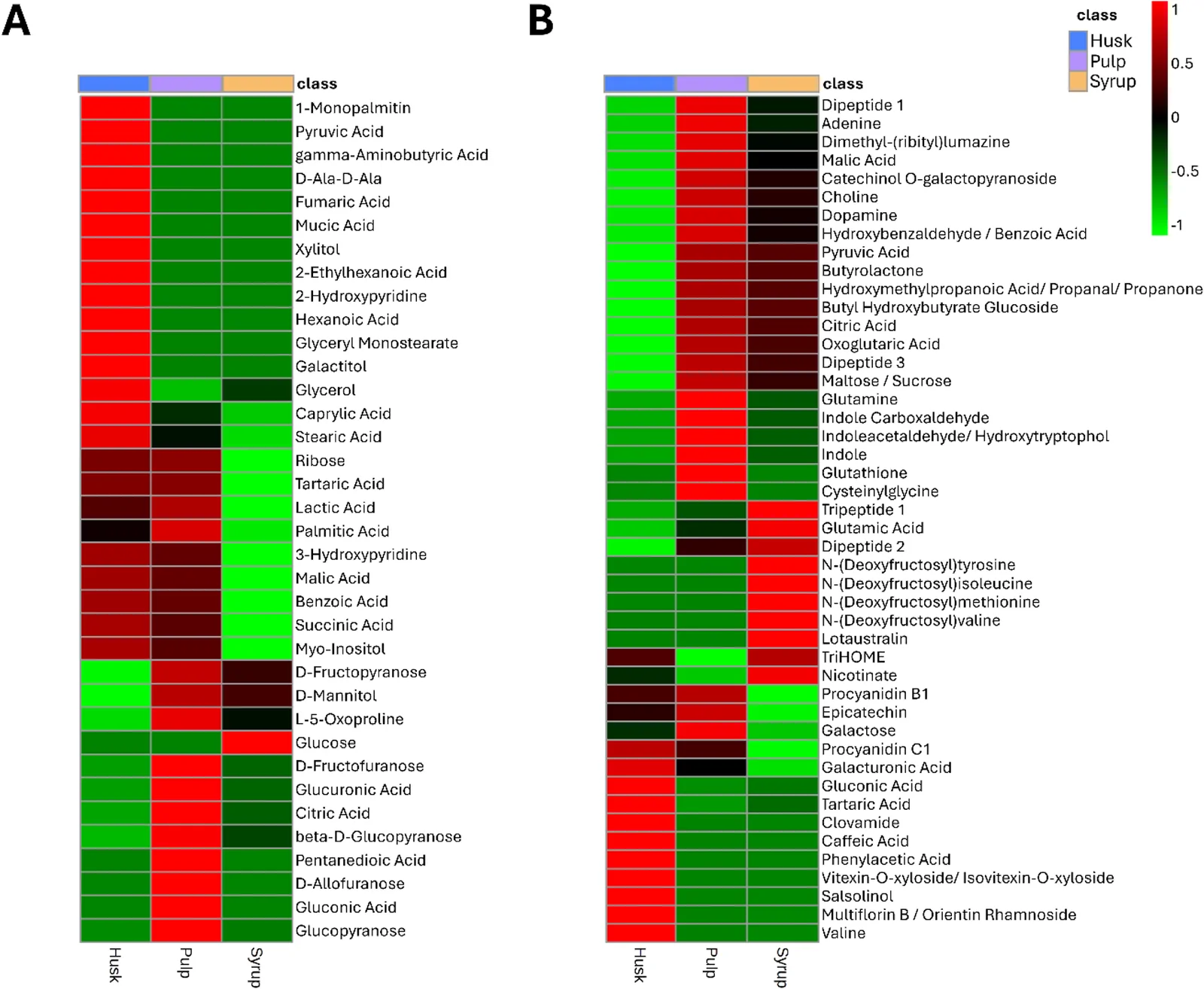
Heatmaps of metabolite profiles across cocoa by-products analyzed by GC-MS (**A**) and LC-MS (**B**). Rows represent individual annotated metabolites, while columns correspond to the analyzed cocoa byproducts (cocoa pod husk, cocoa pulp juice, and cocoa syrup). Both analytical platforms display matrix-specific metabolic signatures across byproducts. The color scale represents autoscaled values of the total area–normalized relative abundances (row-wise z-scores), where red indicates higher, and green lower, relative levels within each metabolite.

In both data sets, cocoa pulp juice, cocoa syrup, and cocoa pod husk displayed matrix-dependent patterns in their annotated metabolite profiles. These patterns were particularly apparent in the LC-MS data set, where structurally complex metabolites showed matrix-specific distributions. In contrast, GC-MS profiles showed broader similarities across matrices, particularly among low-molecular-weight compounds such as organic acids, sugars, and polyols. Notably, cocoa syrup exhibited a metabolic profile with features shared with both pulp-derived and structurally complex matrices, reflecting its origin as a fermentation-derived liquid fraction subjected to subsequent processing. Overall, these results indicate that each cocoa matrix possesses a distinct metabolic profile, shaped by both biological origin and process-driven transformations.

At the compound level, this separation was driven by a small set of matrix-restricted metabolites: Amadori derivatives (N-(deoxyfructosyl)­valine, isoleucine, methionine, and tyrosine) in cocoa syrup; flavan-3-ols and phenolic amides (epicatechin, procyanidins B1 and C1, clovamide, and caffeic acid) in cocoa pod husk; and glutathione, glutamine, cysteinylglycine, and indole-derived compounds in cocoa pulp juice.

Consistent with these matrix-specific profiles, each cocoa byproduct exhibited a distinct metabolomic profile reflecting its biological origin and processing history (Tables 2 and 3). In summary, 82 metabolites were annotated across the three cocoa byproducts, including 46 by LC-MS and 36 by GC-MS. Among the LC-MS annotations, one compound (2.17%) was assigned to identification level 1, 16 compounds (34.78%) to level 2, 25 compounds (54.35%) to level 3, and four compounds (8.70%) to level 4 according to the confidence framework of Blaženović et al.^8^ Caffeic acid was the only level 1 metabolite and was confirmed using an authentic standard. The 36 metabolites annotated by GC-MS were assigned to level 2 based on spectral library matching (match factor >90) supported by retention index agreement.

**Table 2:** Annotated Metabolites Detected in Cocoa Pulp Juice, Cocoa Syrup, and Cocoa Pod Husk by LC-MS Analysis^a^

								Relative Abundance^b^		
Compound Name	Molecular Formula	Molecular Weight (Da)	RT (min)	m/z Error (ppm)	ID Level	DET	Adduct	Cocoa Pulp Juice	Cocoa Syrup	Cocoa Pod Husk
***Amino acids, peptides and analogues***										
Valine	C_5_H_11_NO_2_	117.0790	1.320	8	3	ESI+	[M + H]^+^	_	_	4.91
Glutamine	C_5_H_10_N_2_O_3_	146.0691	1.160	7	2	ESI–	[M – H]^−^	2.09	_	_
Glutamic acid	C_5_H_9_NO_4_	147.0532	2.100	3	3	ESI+	[M + H-H_2_O]^+^	1.79	4.60	_
Cysteinylglycine	C_5_H_10_N_2_O_3_S	178.0412	1.890	1	3	ESI+	[M + H]^+^	1.73	_	_
N-(Deoxyfructosyl)valine	C_11_H_21_NO_7_	279.1318	1.730	3	3	ESI+	[M + H]^+^	_	1.34	_
N-(Deoxyfructosyl)isoleucine	C_12_H_23_NO_7_	293.1475	3.010	3	2	ESI+	[M + H]^+^/[M + H-H_2_O]^+^	_	5.01	_
Glutathione	C_10_H_17_N_3_O_6_S	307.0838	1.880	4	2	ESI+/ESI–	[M + H]^+^	3.71	_	_
N-(Deoxyfructosyl)methionine	C_11_H_21_NO_7_S	311.1039	1.960	2	3	ESI+	[M + H-H_2_O]^+^	_	1.41	_
N-(Deoxyfructosyl)tyrosine	C_15_H_21_NO_8_	343.1267	2.570	2	3	ESI+	[M + H]^+^	_	2.49	_
Adenine	C_5_H_5_N_5_	135.0545	1.730	5	3	ESI+	[M + H]^+^	1.06	_	2.27
Dipeptide 1	C_11_H_16_N_4_O_5_	284.1121	3.500	3	3	ESI–	[M – H-H_2_O]^−^	1.08	_	_
Dipeptide 2	C_14_H_14_N_4_O_6_	334.0913	5.710	7	3	ESI–	[M – H-H_2_O]^−^	_	1.11	1.04
Dipeptide 3	C_13_H_13_N_3_O_9_	355.0652	1.850	2	4	ESI–	[M + Cl]^−^	3.14	2.51	_
Tripeptide 1	C_25_H_24_N_2_O_8_	480.1533	2.610	7	4	ESI–	[M + Cl]^−^	1.10	5.75	_
***Carbohydrates and carbohydrate conjugates***										
Galactose	C_6_H_10_O_6_	178.0477	1.270	7	3	ESI–	[M + HCOOH-H]^−^	_	_	1.83
Galacturonic acid	C_6_H_10_O_7_	194.0427	1.210	2	2	ESI–	[M – H]^−^	_	_	1.27
Gluconic acid	C_6_H_12_O_7_	196.0583	1.180	4	2	ESI–	[M – H]^−^	_	_	2.81
Maltose/Sucrose	C_12_H_22_O_11_	342.1162	1.280	0	3	ESI–	[M – H]^−^	15.41	11.67	1.81
Lotaustralin	C_11_H_19_NO_6_	261.1212	1.730	3	3	ESI+	[M + H]^+^	_	1.01	_
Butyl hydroxybutyrate glucoside	C_14_H_26_O_8_	322.1628	7.620	1	3	ESI–	[M + HCOOH-H]^−^	1.08	1.03	1.34
TriHOME	C_18_H_34_O_5_	330.2406	18.030	2	2	ESI–	[M – H]^−^	_	_	2.86
***Organic acids and phenolic compounds***										
Tartaric acid	C_4_H_6_O_6_	150.0164	1.230	4	2	ESI–	[M – H]^−^	_	_	1.99
Pyruvic acid	C_3_H_4_O_3_	88.0160	2.060	5	3	ESI–	[M – H]^−^	4.36	3.87	_
Malic acid	C_4_H_6_O_5_	134.0215	1.540	8	2	ESI–	[M – H]^−^	4.77	3.50	6.48
Oxoglutaric acid	C_5_H_6_O_5_	146.0215	2.060	6	4	ESI–	[M – H]^−^	2.97	2.52	_
Citric acid	C_6_H_8_O_7_	192.0270	2.060	4	2	ESI–	[M – H]^−^	2.92	2.52	_
Hydroxymethylpropanoic acid/Propanal/Propanone	C_4_H_8_O_3_/C_3_H_6_O	104.0473/58.0419	2.060	2	3	ESI–	[M – H]^−^/ [M + HCOOH-H]^−^	3.78	3.35	1.00
Phenylacetic acid	C_8_H_8_O_2_	136.0524	10.800	3	2	ESI–	[M – H]^−^	_	_	1.16
Clovamide	C_18_H_17_NO_7_	359.1005	10.810	1	3	ESI–	[M – H]^−^	_	_	6.33
Caffeic acid	C_9_H_8_O_4_	180.0423	10.810	2	1	ESI+	[M + H-H_2_O]^+^	_	_	4.27
Hydroxybenzaldehyde/Benzoic acid	C_7_H_6_O_2_	122.0368	2.780	3	3	ESI+	[M + H]^+^	1.19	_	_
Dopamine	C_8_H_11_NO_2_	153.0790	2.780	3	2	ESI+	[M + H-H_2_O]^+^	1.89	1.03	_
***Organoheterocyclic and organonitrogen compounds***										
Butyrolactone	C_4_H_6_O_2_	86.0368	2.060	7	3	ESI–	[M – H]^−^	3.78	3.38	_
Indole	C_8_H_7_N	117.0578	7.000	4	3	ESI+	[M + H]^+^	1.54	_	_
Nicotinate	C_6_H_5_NO_2_	123.0320	1.730	6	3	ESI+	[M + H]^+^	_	_	1.93
Indole carboxaldehyde	C_9_H_7_NO	145.0528	6.990	2	2	ESI+	[M + H]^+^	1.97	_	_
Salsolinol	C_10_H_13_NO_2_	179.0946	2.680	2	2	ESI+	[M + H]^+^	_	_	1.80
Dimethyl-(ribityl)lumazine	C_13_H_18_N_4_O_6_	326.1226	8.800	1	3	ESI–	[M – H-H_2_O]^−^	1.14	_	_
Indoleacetaldehyde/Hydroxytryptophol	C_10_H_9_NO/C_10_H_11_NO_2_	159.0684/177.079	7.000	3	3	ESI+	[M + H]^+^/[M + H-H_2_O]^+^	2.15	_	_
Choline	C_5_H_14_NO	104.1073	1.160	0.68	2	ESI+	[M]^+^	1.36	1.18	6.96
***Polyketides and flavonoids***										
Epicatechin	C_15_H_14_O_6_	290.0790	9.730	0	2	ESI–	[M – H]^−^	_	_	1.49
Catechinol O-galactopyranoside	C_21_H_24_O_12_	468.1268	8.590	8	4	ESI–	[M – H]^−^	2.13	1.42	_
Vitexin-O-xyloside/Isovitexin-O-xyloside/Kaempferol rhamnoside-xyloside	C_26_H_28_O_14_	564.1479	10.670	3	3	ESI+/ESI–	[M + H]^+^	_	_	2.10
Procyanidin B1	C_30_H_26_O_12_	578.1424	9.030	2	2	ESI–/ESI+	[M – H]^−^	_	_	1.93
Multiflorin B/Orientin rhamnoside	C_27_H_30_O_15_	594.1585	9.660	2	3	ESI+	[M + H]^+^	_	_	1.07
Procyanidin C1	C_45_H_38_O_18_	866.2058	10.240	2	3	ESI–	[M – H]^−^	_	_	1.31

a Metabolites are listed along with their molecular formula, database molecular weight , retention time (RT), mass accuracy (m/z Error, ppm), detection mode (DET), identification level (ID level), and detected adducts. Relative abundance values correspond to total area normalized relative abundances (%) within each matrix and are presented for comparative purposes only. Metabolites are organized according to their occurrence across matrices and represent the most abundant and/or confidently annotated compounds detected. Identification levels follow established metabolomics reporting standards, with Level 2 indicating putatively annotated compounds based on spectral similarity and database matching. Blank cells (_) indicate absence or signals below detection threshold.

b Relative abundance was calculated by normalizing the peak area of each feature to the total peak area of the corresponding sample. Only features with relative abundance >1.0% were selected for identification. Values correspond to the mean relative abundance across the three independent biological replicates. “–” indicates that the compound was not detected above the applied selection threshold.

**Table 3:** Metabolites Detected in Cocoa Pulp Juice, Cocoa Syrup, and Cocoa Pod Husk by GC-MS Analysis^a^

					Relative Abundance^b^		
Compound Name	Molecular Formula	Molecular Weight (Da)	RT (min)	Match Factor	Cocoa Pulp Juice	Cocoa syrup	Cocoa Pod Husk
***Alcohol***							
Glycerol	C_3_H_8_O_3_	92.0473	10.037	94.5	_	0.04	0.26
***Amino acids, peptides and analogues***							
Gamma-Aminobutyric Acid	C_4_H_9_NO_2_	103.0633	13.373	100	_	_	0.02
L-5-Oxoproline	C_5_H_7_NO_3_	129.0426	13.280	96.2	0.04	0.05	_
d-Ala-d-Ala	C_6_H_12_N_2_O_3_	160.0848	12.993	100	_	_	0.00
***Carbohydrates and carbohydrate conjugates***							
D-Mannitol	C_6_H_14_O_6_	182.0790	17.666	100	18.39	35.28	22.30
Myo-Inositol	C_6_H_12_O_6_	180.0634	19.526	97.6	5.45	0.11	7.90
Galactitol	C_6_H_14_O_6_	182.0790	17.592	100	_	_	0.33
Ribose	C_5_H_10_O_5_	150.0528	15.015	100	0.05	_	0.06
Xylitol	C_5_H_12_O_5_	152.0685	15.253	100	_	_	0.04
Glucopyranose	C_6_H_12_O_6_	180.0634	18.410	96.7	6.08	1.76	_
Beta-d-Glucopyranose	C_6_H_12_O_6_	180.0634	17.572	96.5	5.09	7.07	_
D-Fructofuranose	C_6_H_12_O_6_	180.0634	16.658	95.4	1.40	0.98	_
D-Allofuranose	C_6_H_12_O_6_	180.0634	17.052	94.1	0.09	_	_
D-Fructopyranose	C_6_H_12_O_6_	180.0634	16.827	93	0.07	0.24	_
Glucose	C_6_H_12_O_6_	180.0634	17.569	95.6	_	0.12	_
***Fatty acyls and lipids***							
Stearic Acid	C_18_H_36_O_2_	284.2715	20.546	100	0.06	_	0.31
Palmitic Acid	C_16_H_32_O_2_	256.2402	18.754	100	0.19	_	0.20
Glyceryl Monostearate	C_21_H_42_O_4_	358.3083	24.827	100	_	_	0.13
1-Monopalmitin	C_19_H_38_O_4_	330.2770	23.416	100	_	_	0.05
Caprylic Acid	C_8_H_16_O_2_	144.1150	9.782	100	0.01	_	0.03
Hexanoic Acid	C_6_H_12_O_2_	116.0837	6.990	100	_	_	0.03
***Organic acids and phenolic compounds***							
Glucuronic Acid	C_6_H_10_O_7_	194.0427	17.390	100	31.11	34.12	32.61
Gluconic Acid	C_6_H_12_O_7_	196.0583	17.495	100	21.91	19.08	23.80
Malic Acid	C_4_H_6_O_5_	134.0215	12.876	94.2	8.07	0.28	10.44
Tartaric Acid	C_4_H_6_O_6_	150.0164	14.792	93.3	0.33	_	0.40
Mucic Acid	C_6_H_10_O_8_	210.0376	18.712	100	_	_	0.32
Lactic Acid	C_3_H_6_O_3_	90.0317	6.868	92.4	0.23	0.03	0.19
Succinic Acid	C_4_H_6_O_4_	118.0266	10.502	100	0.15	_	0.20
Pyruvic Acid	C_3_H_4_O_3_	88.016	6.695	100	_	_	0.21
Fumaric Acid	C_4_H_4_O_4_	116.0110	10.940	100	_	_	0.07
2-Ethylhexanoic Acid	C_8_H_16_O_2_	144.115	8.388	100	_	_	0.03
Citric Acid	C_6_H_8_O_7_	192.027	16.736	93.1	0.99	0.83	_
Pentanedioic Acid	C_5_H_8_O_4_	132.0423	13.915	92.2	0.28	_	_
Benzoic Acid	C_7_H_6_O_2_	122.0368	9.583	100	0.03	_	0.04
***Organoheterocyclic compounds***							
2-Hydroxypyridine	C_5_H_5_NO	95.0371	6.484	100	_	_	0.02
3-Hydroxypyridine	C_5_H_5_NO	95.0371	7.984	100	0.01	_	0.01

a Compounds are listed along with their molecular formula, database molecular weight, and retention time (RT). Relative abundance values correspond to total-area normalized relative abundances (%), within each matrix and are provided for comparative purposes only. The detected metabolites are predominantly low-molecular-weight compounds, including organic acids, sugars, and polyols, which represent key components of primary metabolism and ccarbohydrate transformation pathways, and metabolites are organized according to their occurrence and relative abundance across matrices. Blank cells (_) indicate absence or signals below the detection threshold .

b The relative abundance was calculated by normalizing the peak area of each feature to the total peak area of the corresponding sample.

Experimental MS/MS spectra of selected levels 1–2 LC-MS metabolites are provided in Supplementary Figure S1. At the same time, a subset of metabolites was consistently detected across matrices, indicating the persistence of shared biochemical pools throughout the cocoa processing chain (Figure 2).

Cocoa pulp juice, corresponding to the mucilaginous liquid fraction surrounding the seeds prior to fermentation, was primarily associated with metabolites characteristic of fresh plant tissues. LC-MS analysis (Table 2) revealed organic acids linked to central metabolism, including malic, citric, pyruvic, and oxoglutaric acid,^38^ together with amino acids and small peptides such as glutamine, glutamic acid, and cysteinylglycine. Reduced sulfur-containing metabolites, including glutathione, as well as indole-derived compounds, were also detected. Complementary GC-MS data (Table 3) showed a profile dominated by sugar-derived acids and polyols, particularly glucuronic acid, gluconic acid, and mannitol, along with multiple glucose and fructose isomers. This composition is consistent with a soluble fraction derived directly from fresh pulp tissues prior to microbial transformation.^39^

In contrast, cocoa syrup displayed a metabolomic profile shaped by both microbial activity during fermentation and subsequent processing steps, including concentration and thermal stabilization. LC-MS analysis (Table 2) revealed soluble carbohydrates such as maltose and sucrose and organic acids including malic and citric acids, together with multiple N-(deoxyfructosyl) amino acid derivatives, such as N-(deoxyfructosyl)­valine and N-(deoxyfructosyl)­isoleucine. These Amadori compounds were detected in cocoa syrup but were not detected in either the prefermentation pulp juice or cocoa pod husk generated within the same production system (Table 2), including derivatives annotated at identification level 2. Amadori compounds are recognized as early-stage Maillard reaction products arising from reactions between reducing sugars and amino compounds, and their detection in the processed syrup is consistent with chemical transformations occurring during the processing history of this matrix.^40,41^ Together with the distinct mineral profile described above, these findings provide compositional evidence that the cocoa syrup investigated here constitutes a distinct processed matrix rather than being compositionally interchangeable with the unprocessed liquid cocoa fractions described in the literature. Nitrogen-containing metabolites such as choline and related compounds were also detected. GC-MS data (Table 3) further indicated high abundances of polyols and carbohydrate-derived acids, particularly mannitol, glucuronic acid, and gluconic acid. Compared to the other matrices, cocoa syrup exhibited a lower diversity of lipophilic compounds, consistent with its liquid nature. Overall, this fraction represents a compositionally distinct intermediate integrating pulp-derived compounds with fermentation- and processing-derived metabolites.

Cocoa pod husk, representing the outer pericarp of the fruit, exhibited a markedly different metabolomic profile consistent with a structurally complex plant matrix. LC-MS analysis (Table 2) revealed phenolic compounds and flavonoids characteristic of cocoa tissues, including epicatechin, clovamide, and procyanidins (B1 and C1), as well as flavones.^42^ Organic acids and amino acids were also detected, although their relative distribution differed from that of the pulp-derived fractions. GC-MS analysis (Table 3) showed comparatively lower relative abundances of simple sugars and a greater representation of organic acids, fatty acids (such as palmitic and stearic acids), and structural metabolites, including myo-inositol. This composition is consistent with lignocellulosic plant tissues, in which structural and phenolic components dominate the chemical profile.^23^ The lignocellulosic fraction of cocoa pod husk has been extensively characterized elsewhere, with reported contents of cellulose, hemicellulose, lignin, and pectin, making it a recognized source of dietary fiber, including pectin.^11,23,33^ That fraction was not quantified here and is complementary to the soluble metabolite pool described in this work; together, both dimensions would provide a more comprehensive compositional basis for assessing the valorization potential of this matrix.

Beyond these matrix-specific signatures, several metabolites were consistently detected across multiple matrices, reinforcing the presence of shared biochemical pathways within the cocoa processing system. In the LC-MS data set (Table 2), compounds such as choline and malic acid were detected in all three matrices, while central metabolites including pyruvic acid, oxoglutaric acid, citric acid, glutamic acid, and disaccharides such as maltose/sucrose were shared between cocoa pulp juice and cocoa syrup. Similarly, GC-MS analysis (Table 3) revealed conserved sugar-derived acids and polyols across matrices, particularly glucuronic acid, gluconic acid, and mannitol, which were consistently detected across all three analyzed byproducts. The persistence of these compounds suggests continuity of carbohydrate-derived metabolic pools along the cocoa processing chain. This study indicates that cocoa byproducts are characterized by both distinct and shared metabolomic features, suggesting the interplay between biological origin and process-driven transformations. While the relative abundance profiles capture the global organization of this metabolic space, compound-level annotation provides insight into the specific biochemical patterns underlying these profiles.

### Matrix-Resolved Distribution of Annotated Metabolites: A Preliminary Framework for Prioritizing Future Valorization Studies

To organize the annotated compounds in a way that can guide future research, a subset of metabolites detected in the LC-MS and GC-MS data sets was selected based on annotation confidence. To minimize overinterpretation of putatively annotated compounds, this analysis was restricted to metabolites assigned to identification levels 1 or 2 (Table 4).

**Table 4:** Selected Metabolites Detected in Cocoa By-Products and Their Applications Reported in the Literature^a^,^b^

			*Matrix Occurrence*			
*Chemical Group*	*Molecule*	Applications Reported in the Literature	*Cocoa Pulp Juice*	*Cocoa Syrup*	*Cocoa Pod Husk*	*References*
Sugars and Polyols	D-Mannitol	Used as a carbon source or feedstock in microbial biomanufacturing processes for value-added products.	●	●	●	^43^
	β-d-Glucopyranose	Used as a substrate in enzymatic and microbial processes, including applications involving β-glucosidases and biopolymer conversion.	●	●	○	^44^
	Myo-Inositol	Used in nutraceutical, pharmaceutical, and food formulations and investigated in relation to insulin sensitivity and metabolic and hormonal disorders.	●	●	●	^45^
	D-Fructofuranose	Used as a fructosyl donor in enzymatic fructooligosaccharide (FOS) synthesis for food and nutraceutical applications.	●	●	○	^46^
Organic Acids	Glucuronic acid	Biosynthetic precursor for glycosaminoglycans and intermediate in routes toward glucaric acid and other biobased products.	●	●	●	^47,48^
	Gluconic acid	Used as an acidulant, chelating agent, and mineral carrier in food, pharmaceutical, and other industrial applications.	●	●	●	^49^
	Malic acid	Used as an acidulant in food and beverages and investigated as a biobased platform chemical for polymer production.	●	●	●	^50^
	Citric acid	Widely used as an acidulant, preservative, and chelating agent in food, beverage, pharmaceutical, and cosmetic applications.	●	●	○	^51^
	Tartaric acid	Used as an acidulant and stabilizer and as a chiral building block in chemical and pharmaceutical synthesis.	●	○	●	^52^
Phenolics and Polyphenols	Caffeic acid	Investigated for antioxidant and anti-inflammatory activities and for applications in food, cosmetic, and biomedical research.	○	○	●	^53^
	Procyanidin B1	Investigated for antioxidant, anti-inflammatory, cardiometabolic, and gut microbiota-related activities in functional food and nutraceutical research.	○	○	●	^54^
	Epicatechin	Investigated for cardiovascular and neuroprotective activities, including effects on endothelial and cellular signaling pathways.	○	○	●	^55^
	Dopamine	Investigated for antioxidant and stress-related functions in plants and as a precursor of polydopamine materials for antimicrobial and antioxidant coatings.	●	●	○	^56^
Amino Acids and Derivates	Glutathione	Investigated for its role in cellular redox homeostasis, oxidative-stress regulation, and related biomedical and nutritional applications.	●	○	○	^57^
	Glutamine	Used in clinical nutrition and investigated in relation to intestinal barrier function, immune responses, and gut microbiota modulation.	●	○	○	^58^
	Choline	Used in nutritional supplementation and investigated in relation to neurodevelopment, liver function, and cardiometabolic health.	●	●	●	^59^
	N-(Deoxyfruct.)isoleucine	Amadori rearrangement product investigated for metal-chelating, antioxidant, and digestive enzyme–inhibitory activities.	○	●	○	^60^
Lipids and Fatty Acids	TriHOME	Investigated for isomer-dependent roles in inflammatory and barrier-related processes and as a potential biomarker in metabolic and respiratory research.	○	○	●	^61^
	Palmitic acid	Investigated for its roles in lipid signaling, S-palmitoylation, innate immune responses, and inflammation-related processes.	●	○	●	^62^
	Stearic acid	Investigated for effects on lipid metabolism, mitochondrial dynamics, and metabolic and gut microbiota-related processes.	●	○	●	^63^
Indoles and Alkaloids	Indole carboxaldehyde	Investigated as an aryl hydrocarbon receptor (AhR) ligand in relation to intestinal barrier, inflammatory, and gut–brain-axis processes.	●	○	○	^64^
	Salsolinol	Investigated for concentration- and stereoisomer-dependent neurobiological effects and its involvement in dopaminergic pathways.	○	○	●	^65^

a Metabolites detected by LC-MS and GC-MS were Restricted to compounds assigned to identification levels 1 or 2 and are grouped according to chemical class, and matrix occurrence indicates detection in cocoa pulp juice, cocoa syrup, and cocoa pod husk. Reported applications were compiled from previous studies and are included to contextualize potential directions for subsequent research; these applications were not evaluated in the present study, and the table therefore provides a qualitative mapping between compound identity , matrix occurrence , and previously reported applications and should not be interpreted as evidence of metabolite concentration , recoverability, functional activity , or industrial feasibility in the cocoa matrices investigated here.

b *(● = detected/○ = not detected).* Applications listed were compiled from published studies performed with purified compounds or matrices other than the cocoa by-products investigated here and were not evaluated in the present study. Matrix occurrence (●) indicates detection only and does not imply concentrations sufficient for recovery, functional activity, or industrial applicability.

The resulting compilation is intended as a hypothesis-generating framework linking compound identity and matrix occurrence to potential directions for subsequent experimental investigation.

The selected compounds span diverse chemical groups, including organic acids, polyols, amino acid derivatives, phenolic compounds, lipids, and Maillard-related products, many of which have been investigated in food, nutraceutical, cosmetic, and biotechnological contexts, generally using purified compounds or source materials unrelated to cocoa.^66–68^ Rather than aiming for exhaustive classification, Table 4 highlights selected compounds together with applications previously reported in the literature, providing a first-level mapping between metabolite identity, matrix occurrence, and possible directions for future investigation. Importantly, these compounds showed distinct occurrence patterns across the three cocoa matrices. Core metabolites associated with primary metabolism (e.g., sugars, polyols, and central organic acids) were broadly distributed across matrices, whereas phenolic compounds and complex lipids were predominantly detected in the cocoa pod husk. In contrast, fermentation- and processing-derived compounds, including Maillard-related products, were primarily detected in the cocoa syrup. This matrix-resolved distribution provides an additional descriptive layer beyond the compound annotation. Within the analytical scope of this study, cocoa pulp juice was characterized predominantly by soluble primary metabolites, cocoa syrup by soluble and process-derived compounds, and cocoa pod husk by a metabolite profile with a notable representation of phenolic compounds.

## Scope and Limitations

This study is descriptive: it establishes which compound classes predominate in each stream, not how much of each is present or how it behaves. Absolute quantification, extraction yields, and bioactivity assays were outside its scope, and the applications compiled in Table 4 are those reported in the literature for each compound in other contexts; the presence of a metabolite does not predict its recoverability, stability, or functional performance, which are governed by matrix effects and compound interactions.^69,70^ The proximate and mineral data derive from batch-composite samples analyzed in triplicate, a design that yields an integrated reference value per matrix but does not support inferential comparison between by-products; establishing the statistical significance of the differences described here will require replication across independent batches, seasons, and clones. Comparisons with published values are contextual rather than quantitative, since reported compositions differ in their basis of expression and in postharvest conditions. Moisture, dietary fiber, and potentially toxic elements were not determined, so this work does not constitute a safety assessment, and any food application would require prior verification of compliance with regulatory limits (a determination that is particularly critical for a concentrated fraction, such as the syrup). Finally, these results describe cocoa originating from a single geographic location and processed within one specific setup: industrial-scale monoclonal fermentation in wooden boxes under a certified quality management system. Therefore, the compositional profiles reported here should not be directly extrapolated to cocoa from other geographic origins or production systems. Less standardized systems, where fermentation may occur in direct contact with the ground or in heterogeneous containers, are also likely to yield different mineral profiles.

Within these limits, the data set defines a specific experimental agenda: absolute quantification of the prioritized compounds against authentic standards, determination of total, soluble, and insoluble dietary fiber in cocoa pod husk, systematic comparison of extraction strategies with yield and purity determination, quantification of potentially toxic elements, and validation of functional properties under application-relevant conditions.

## Conclusions

The three by-products generated along the on-farm fine-flavor cocoa chain share a comparable proximate composition but diverge sharply in mineral content and metabolite profiles. Cocoa syrup emerged as a mineral-enriched fraction whose enrichment relative to its own prefermentation precursor is selective rather than purely concentrative. Among the 82 metabolites annotated, each matrix showed a distinctive signature: glutamine, cysteinylglycine, glutathione, and indole derivatives in cocoa pulp juice; Amadori derivatives in cocoa syrup; and clovamide, caffeic acid, epicatechin, and procyanidins B1 and C1 in cocoa pod husks.

The main contribution of this work lies in the comparative analysis of three coexisting byproducts within the same production system and under a single analytical protocol. This design also provides a basis for distinguishing compounds associated with the raw material from those associated with processing, an interpretation that is more difficult to make when a derived product is characterized in isolation. This includes, to our knowledge, the first joint compositional and metabolomic description of a thermally concentrated, fermentation-derived cocoa syrup together with prefermentation cocoa pulp juice recovered from the same production batches, two liquid materials that are frequently grouped under overlapping terminology in the literature.

In practice, these profiles provide a baseline for prioritizing byproducts and candidate compounds for subsequent investigation: phenolic compounds and selected lipids point to cocoa pod husks, soluble primary metabolites to cocoa pulp juice, and process-derived compounds to cocoa syrup. Because all three matrices were characterized using a single analytical protocol, this mapping provides a basis for selecting candidate source matrices for subsequent targeted studies without relying exclusively on comparisons across independent studies. This prioritization is intended to serve as a starting point for hypothesis-driven research rather than as a demonstration of industrial viability or suitability for food use.

## Supplementary Material



## Data Availability

All data generated or analyzed during this study are included in this published article. The raw LC-MS and GC-MS data sets generated are publicly available in MassIVE. LC-MS data are available at ftp://massive-ftp.ucsd.edu/v14/MSV000102746/, while GC-MS data are deposited at ftp://massive-ftp.ucsd.edu/v14/MSV000102747/.

## Notes

Analytical repeatability data for proximate and mineral determinations (Table S1); experimental MS/MS spectra of selected metabolites annotated by LC-MS/MS (Figure S1) (PDF)
