# Supplementary material for: Compositional and Metabolomic Characterization of Major On-Farm Side Streams and Derived Products of Industrial Fine-Flavor Cocoa Processing
Source: ACS Omega. 2026 Sep 30;11(40):61241–55. doi: 10.1021/acsomega.6c06084 (PMC13670318; doi:10.1021/acsomega.6c06084)
Supplement: Supplementary file 1 [file ao6c06084_si_001.pdf]

COMPOSITIONAL AND METABOLOMIC CHARACTERIZATION OF MAJOR ON-FARM SIDE STREAMS AND DERIVED PRODUCTS OF INDUSTRIAL FINE-FLAVOR COCOA PROCESSING

Daniela Burgos-Toro, Ana M. León-Inga, María J. Chica, José M. Guillamón, Mónica P. Cala, Miguel Fernández-Niño

SUPPLEMENTARY MATERIAL

Supplementary Table S1. Analytical repeatability (intra-laboratory precision) of the proximate and mineral determinations performed on the three on-farm cocoa by-products. Values correspond to three replicate analytical determinations (n = 3) performed by LASEREX (Universidad del Tolima, Colombia) on a single composite sample per by-product, prepared by homogenizing equal amounts of material from three independent industrial production batches. CV = coefficient of variation, calculated as (SD / mean) × 100. Crude protein was determined by the Kjeldahl method; ash and ether extract by gravimetric methods; Ca, Mg, Na, K, Fe, Cu, Mn, and Zn by atomic absorption spectrophotometry; and B, P, and S by UV–Vis spectrophotometry, following AOAC procedures.

Parameter	Cocoa pod husk		Cocoa pulp juice		Cocoa syrup	
	Mean ± SD	CV (%)	Mean ± SD	CV (%)	Mean ± SD	CV (%)
Crude protein (%)	1.223 ± 0.023	1.89	0.860 ± 0.010	1.16	1.257 ± 0.031	2.43
Ether extract (%)	0.307 ± 0.006	1.88	0.024 ± 0.001	4.17	0.110 ± 0.010	9.09
Ash (%)	2.010 ± 0.026	1.32	0.433 ± 0.025	5.81	5.383 ± 0.012	0.21
Calcium (%)	1.020 ± 0.010	0.98	0.058 ± 0.002	2.65	0.253 ± 0.006	2.28
Potassium (%)	0.450 ± 0.010	2.22	0.092 ± 0.003	3.15	1.257 ± 0.032	2.56
Magnesium (%)	0.049 ± 0.001	1.19	0.015 ± 0.001	7.53	0.083 ± 0.002	2.09
Phosphorus (%)	0.044 ± 0.001	2.27	0.010 ± 0.000	<1	0.035 ± 0.001	1.63
Sulfur (%)	0.023 ± 0.001	2.55	0.010 ± 0.000	<1	0.026 ± 0.001	3.85
Sodium (mg/kg)	107.83 ± 1.02	0.95	38.30 ± 0.10	0.26	364.76 ± 0.90	0.25
Boron (mg/kg)	79.03 ± 0.96	1.22	6.64 ± 0.24	3.62	44.30 ± 0.36	0.81
Iron (mg/kg)	58.40 ± 0.20	0.34	3.11 ± 0.08	2.69	29.80 ± 1.56	5.24
Manganese (mg/kg)	38.90 ± 0.10	0.26	3.67 ± 0.01	0.27	15.54 ± 0.46	2.94
Zinc (mg/kg)	27.03 ± 0.21	0.77	4.52 ± 0.01	0.26	17.59 ± 0.04	0.25
Copper (mg/kg)	13.13 ± 0.06	0.44	1.73 ± 0.04	2.52	5.66 ± 0.01	0.10

Across the 42 determinations (14 parameters × 3 matrices), coefficients of variation ranged from <1% to 9.09%, with a mean of 2.05% and a median of 1.76%. All determinations showed CV ≤10%, and 38 of 42 (90.5%) showed CV ≤5%. Considering mineral determinations only (33 values), CVs ranged from <1% to 7.53%, with a mean of 1.76%, and 31 of 33 (93.9%) showed CV ≤5%. The highest CVs corresponded to ether extract in cocoa syrup (9.09%) and magnesium in cocoa pulp juice (7.53%).

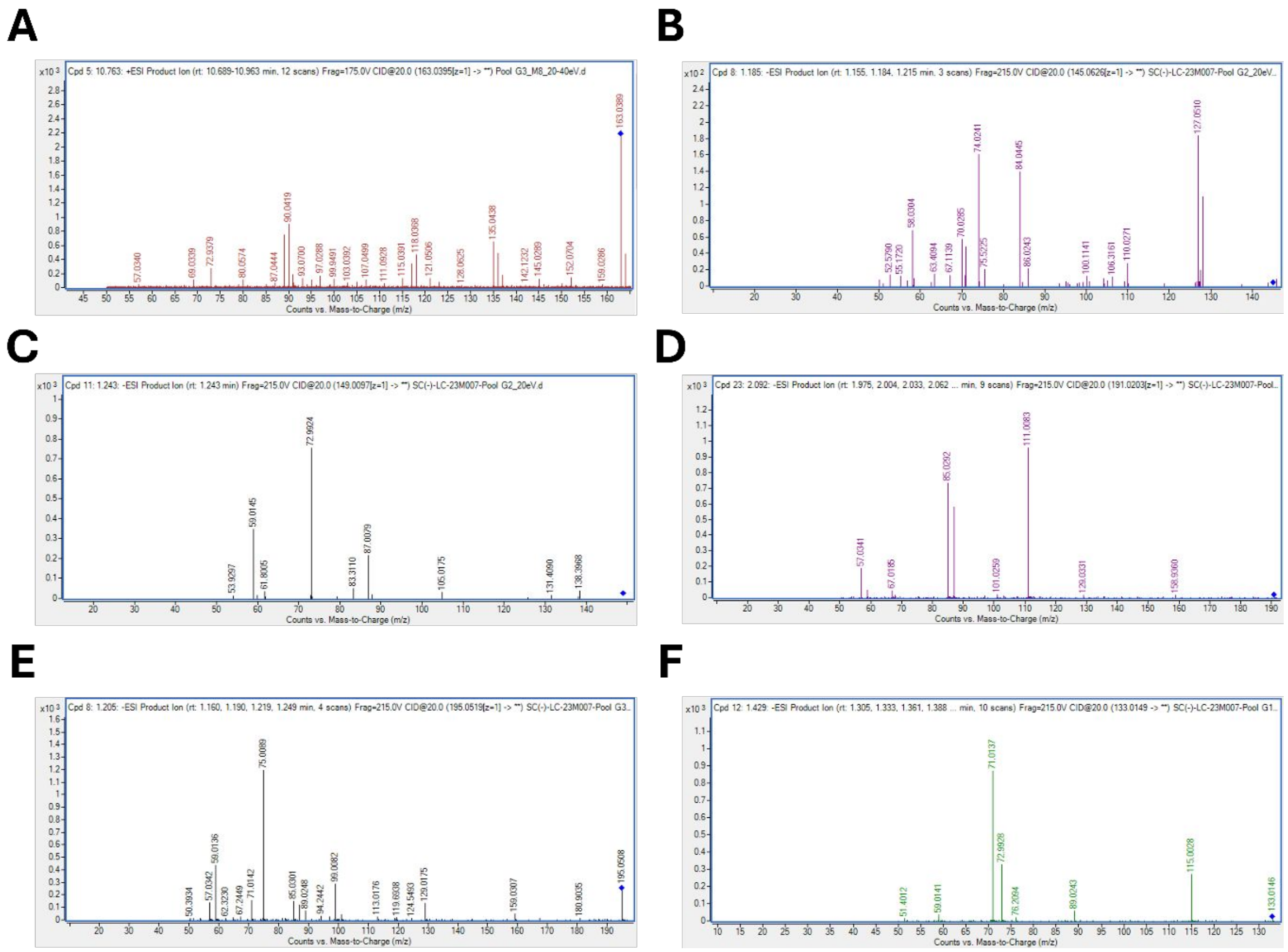

Supplementary Figure S1. Experimental MS/MS spectra of selected metabolites annotated by LC–MS/MS in cocoa on-farm by-products. Caffeic acid **(A)**, assigned to identification level 1, was confirmed using an authentic standard analyzed under the same experimental conditions. Compounds B–F were assigned to identification level 2 based on accurate mass, molecular formula, and MS/MS fragmentation evidence. **(A)** Caffeic acid; **(B)** glutamine; **(C)** tartaric acid; **(D)** citric acid; **(E)** gluconic acid; **(F)** malic acid.
